# A Practical Treatise on Diseases of the Skin

**Published:** 1853-09

**Authors:** 


### REVIEWS.

---

Art. IV.—*A Practical Treatise on Diseases of the Skin.* By J. Moore Neligan, M.D., M.R.I.A., Honorary Fellow of the Society of Physicians of London, Physician to the Jarvis Street Hospital, and Lecturer on Practice of Medicine in the Dublin School of Medicine. Philadelphia: Blanchard & Lea, 1852. 12mo., pp. 333.

There are many reasons why diseases of the skin should interest the practitioner and student of medicine. In the first place, there are none as to which more difficulty is experienced in the diagnosis; and in the second, few resist more obstinately the remedies that have heretofore been addressed to them. Hardly a physician lives, perhaps, who has not at times been puzzled to make out the character of some case of cutaneous disease. Most physicians have probably been troubled in that way very often These complaints constitute a special study, and it is only by long familiarity with them, and careful investigation of their peculiar pathology, that even a moderate degree of knowledge of them can be obtained.

The skin is a most important organ. It is the seat of many of the senses — not of touch merely, but of smelling, tasting, and hearing, and disease of it impairs all these faculties. Disease of the skin is a frequent cause of deafness. Covering the entire body, it has many and important uses in the animal economy, and impairment of its functions can hardly fail to bring on disorder of the general system. The skin being a continuation of the mucous membranes, in its derangements the stomach, the bowels, or the lungs, are apt to become more or less engaged.

This class of diseases, upon which there still rests so much obscurity, has been as long known as any other of the maladies of our race. We know how one of them—the Leprosy — was dreaded by the Jews; and of another — Elephantiasis — we have accounts in the writings of the early Greek and Arabian physicians. Why so little progress has been made in their study does not seem easy to understand. They are external, palpable, exposed to the eye, and would seem to afford every requisite for successful investigation. The microscope has lately been used for the discovery of their true nature, and much was expected from the employment of this instrument, but disappointment to a great extent has followed this expectation. But little addition has yet been made by the microscope to our knowledge of the diseased conditions of the skin. This is particularly true of their diagnosis and treatment, as based on minute anatomy, the very points upon which an increase of knowledge is most to be desired. But this mode of investigation has not been wholly unproductive; some interesting results have followed. It has revealed to us, for example, that in some diseases of the skin a vegetable production—a cryptogamous plant — is present on the cutaneous surface, which is, without doubt, intimately connected with their true pathology. The microscope has disclosed to us a new world—a fauna and flora upon the animal body, as well as within it; and proved that man is literally, what he has often been figuratively styled, a microcosm. Whether these parasites of the skin are the cause or the consequence of the cutaneous affections, is a question not yet settled among pathologists — whether they are a *propter hoc* or a *post hoc;* but as to the existence of these fungi all dermatologists, we believe, are fully persuaded. In other affections of the skin it has been proved, by the aid of glasses, that microscopic animals are present and probably the cause of the disease.

Dr. Neligan omits eruptive fevers from the catalogue of diseases of the skin, and wisely, in our opinion. They are not diseases of the skin, but of the general system — true constitutional diseases, of which the alteration in the skin is merely a symptom, and one of secondary importance. It is opposed to the advanced position of modern pathology, and calculated to keep up incorrect ideas of their essential nature, to include plague, measles, and scarlatina, among the diseases of the skin. He divides these affections into ten groups or orders, as follow —

| | |
|---|---|
| 1. Exanthemata. | 6. Hypertrophiæ. |
| 2. Vesiculæ. | 7. Hemorrhagiæ |
| 3. Pustulæ. | 8. Maculæ. |
| 4. Papulæ. | 9. Cancrodes. |
| 5. Squamæ. | 10. Dermatophytæ. |

Adding two supplementary groups, Syphilides and Diseases of the Appendages of the Skin.

In the following quotation will be found the definition of these several orders —

The diseases contained in the Order Exanthemata are characterized by the occurrence, on a greater or less extended surface of the skin, of a blush of inflammatory redness, usually more or less elevated, which terminates in epidemic desquamation, in the form of fine mealy scales. The most essential character of the order is, that the redness momentarily disappears on pressure with the finger. In one form of eruption, classed amongst the exanthemata, namely, erysipelas, the epidermis is very commonly raised in large blebs by serous effusion; but this is evidently caused by the intensity of the inflammation which is present, takes place in the progress of the disease, and is not a constant or essential symptom. In general, the redness is in large uncircumscribed patches, or uninterruptedly diffused over the surface; but in some forms it occurs in well-defined, regular spots. The essential nature of the exanthemata is, that they are inflammatory, and they seem to have their seat in the vascular rete of the derma. This order corresponds with the second subgroup of the first proup of the first secondary division of Wilson's Natural System, defined by him as "Inflammation of the derma, without constitutional symptoms of a specific kind." It contains four genera, erythema, erysipelas, urticaria, roseola.

The Vesiculæ are characterized by an eruption of vesicles or blebs, which consist in an elevation of the epidermis, varying in size, in some forms minute (vesicles,) and in some of tolerable magnitude (bullæ or blebs,) containing a transparent, serous fluid, which, with the progress of the disease, becomes opaque, and dries into thin scales or hard crusts. The fluid by which the elevation of the epidermis is caused in the vesiculæ is at first transparent and albuminous, but after a short time becomes opaque, and often puriform. This order nearly corresponds with the second group of the first secondary division of Wilson, which he defines as "Effusive Inflammation of the Derma." It contains five genera—eczema, herpes, pemphigus, rupia, scabies. Wilson places scabies in a distinct subgroup, defining it to be "Inflammation of the derma, from the presence of acari."

The third order, Pustulæ, is characterized by the eruption of *Pustules*—rounded elevations of the epidermis containing pus, which, bursting, form scabs or thick crusts. The pustules may be of small size, and closely aggregated together, or large and isolated; the former constituting the *Psydracia*, the latter, the *Phlyzacia*, of Willan; a psydracious pustule being defined by him to be "a minute pustule, irregularly circumscribed, producing but a slight elevation of the cuticle, and terminating in a laminated scab," and a phlyzacious one, "of a larger size, raised on a hard, circular base of a vivid red color, and succeeded by a thick, hard, dark-colored scab." The epidermic covering of a pustule is much thicker than that of a vescicle, consequently it takes a longer time to maturate, or, in other words, to burst and form a scab or crust, but in their advanced stage the diagnosis between the two is sometimes not unattended with difficulty. The eruptions placed in this order correspond with those classed by Wilson in the third group of his first secondary division: "Suppurative Inflammation of the Derma." The genera are three in number: acne, impetigo, ecthyma. Wilson, however, puts acne in a group, the definition of which that he gives being, "Inflammation of the glands and adjacent textures."

The Papulæ are characterized by an eruption of minute, solid elevations—*pimples*—generally reddish, but sometimes of the natural color of the skin, containing neither serum nor pus, terminating in the disquamation of fine scales, and almost invariably attended with intolerable itching. In some forms, the top of the pimple is of a reddish brown or black color, but this merely depends on the accidental presence of a small, dried crust of blood, usually effused by scratching. This order contains two genera: lichen, prurigo. It corresponds with the fourth group of Wilson's first secondary division, which he defines: "Depositive inflammation of the derma."

The eruptive diseases contained in the order Squamæ, are characterized by the secretion of dry, laminated, whitish scales on the cutaneous surface, usually occurring in patches, often of a circular form. The scales, which are somewhat elevated above the level of the skin, readily fall off, to be again rapidly renewed; the part which they cover is of a smooth, glistening aspect, reddish and dry. The order corresponds with the fifth group of the first secondary division of Wilson's classification: "Squamous inflammation of the derma." It contains two genera: psoriasis, pityriasis.

In the order Hypertrophiæ, I include those diseases which are characterised by an hypertrophied condition of the derma or epidermis, or of both, or of the hair follicles. The term has been used much in this sense by Simon, in his "Anatomical Description of Diseases of the Skin," and from him I have adopted it. In its application it is nearly synonymous with the tuberculæ of former artificial systems of classification. The latter term, although probably not so faulty, when first employed to designate a group of cutaneous diseases, is, I think, highly objectionable at present, when it is invariably understood to designate a peculiar morbid deposit, and its application in any other sense must tend to cause confusion. Wilson, in his Natural System, has a group in which are placed those affections that consist in an "hypertrophied state of the papillæ of the derma;" but I propose to extend the application of the term, and to include in the order those diseases in which the hypertrophy affects the other cutaneous structures. I shall place in it nine genera: icthyosis, molluscum, stearrhœa, elephantiasis, verrucæ, clavus, callositates, condylomata, nævi.

The characteristics of the seventh order, Hemorrhagæ, scarcely require to be defined. In it there is a morbid alteration of the capillary circulation, accompanied by a changed or diseased condition of the blood, in which this fluid, escaping from its proper vessels, is extravasated in rounded spots or patches beneath the epidermis, and also beneath the epithelium of the mucous and serous membranes. Bursting through the latter finer structure, more or less bleeding usually takes place from the surfaces of both these membranes. It contains but one genus: purpura.

The order Maculæ is characterised by an alteration in the color of the skin, occurring usually in large patches, and unattended with any eruption. The natural color may be deepened, diminished, or altered in hue, and the affection has its seat evidently in the apparatus of the skin which secretes the pigmentary matter. The order corresponds with the fifth secondary division of Wilson: "Disorders of the Chromatogenous Function of the Derma." It contains two genera: vitiligo, which includes albinoismus, and ephelis.

The order Cancrodes contains those diseases of the skin in which many of their features resemble cancerous affections. They are characterized by a degree of semi-malignancy usually attended with foul ulceration of a slow and insiduous nature, often with severe stinging pain, and a marked tendency to return after apparent cure, or after excision, in the same or in remote parts of the cutaneous surface. It contains two genera: lupus, keloïs.

The tenth order, Dermatophytæ, I have adopted from Bennett. It includes those diseases of the skin which depend on, or are characterized by the presence of, parasitic plants. It containes two genera : porrigo, sycosis.

Of the two supplementary orders, the first—Syphilides—contains those eruptions of the skin which are ordinarily termed *secondary*, being caused by, or consequent on the introduction of the venereal virus or poison into the system ; and the second includes diseased conditions of the *hair* and *nails*.

By the ancients, the term Exanthemate was understood to designate every variety of eruption of the skin. At the present time, the group of diseases included under it is one well defined, and limited ; "they are characterized by the sudden appearance, on a greater or less extended portion of the skin, of an inflammatory redness in variously shaped patches, which momentarily disappear on pressure, are usually attended with a slight elevation of the surface, and terminate in exfoliation of the epidermis." They are almost always attended by inflammatory fever, thus constituting a *natural* group. But, as justly remarked by Dr. Neligan, we should be careful in the treatment of them, not to let the idea of inflammation lead us to too active measures. The fever, instead of antiphlogistic treatment, may be of a low asthenic form, requiring the opposite course.

Erythema is one of the mildest of this group of complaints. Many of the cases which pass as erysipelas are of this character. The diagnosis is generally easy —

The blush of erythema is of a deeper red, and less livid than that of erysipelas, is not attended with the same amount of local tumefaction, burning heat, and pain, and is marked by much less disturbance of the system generally, and much less fever. That form of erythema simplex, which has been named fugax, has sometimes been mistaken for urticaria evanescens ; they are both exanthematous eruptions ; but in the latter the eruption disappears and re-appears with constant rapidity, generally in the same places, while erythema fugax, though nearly equally evanescent, does not return to the same parts; it is, too, unattended with the acute itching and annoying tingling so characteristic of urticaria, and moreover occurs in the course of some disease of the general system, while urticaria evanescens is an idiopathic affection. Erythema papulatum has sometimes been mistaken for some of the eruptive fevers, as for measles or scarlatina; but its local character, its appearance upon the hands, and its papular elevation, serve to distinguish it from measles, in which the eruption is of a crescentic form, of a duller red color, and attended with a catarrhal fever. As regards scarlatina, the bright redness of the efflorescence and the acute inflammatory fever, together with the sore throat of that disease, should suffice to render the diagnosis easy. Intertrigo may be confounded with chronic eczema, occurring behind the ears, in the axillæ, or in the groins; the latter, however, is a vesicular eruption in its primary stage, and as it advances is marked by the copious serous discharge, tumefaction, and deep red fissures, which so remarkably characterize the disease.

Erysipelas, another of the exanthems, is a disease of great interest. It is occasionally a most formidable disease. We have had it repeatedly as an epidemic in our country, within a few years, and the minds of professional men are singularly divided in regard to its treatment. What is the most successful mode of combating erysipelas? We venture to say few more perplexing questions could be propounded. We give what Dr. Neligan has to say on this point —

The treatment of idiopathic erysipelas may be conveniently considered under two heads — the constitutional and the local. As regards the former, many different views have been propounded, the supposed indications depending on the idea formed as to the essential nature of the disease. Thus those who believe it to depend on some deranged condition of the hepatic secretion—the prevalent opinion among them being that it is caused by a deficient secretion of bile and a consequent accumulation of it in the system—treat all forms of erysipelas, no matter what may be the age or condition of the patient, by the administration of remedies calculated to promote a copious evacuation of that fluid by the alimentary canal. Others, regarding it as a highly inflammatory disease, employ depletion, and other active antiphlogistics; but this plan of treatment, although it may succeed in the robust dwellers in country districts, is not at all suited for the inhabitants of town or large cities. With some, the affection being viewed as one of asthenia, being attended with or dependent on diminished vital power, the use of tonics and stimulants is relied upon; while again, others, finding that the urine is highly acid in the early stages of the disease, as it is in all febrile affections, recommend an alkaline treatment.

Seeing that these so opposite plans of treating erysipelas are reported by those who have proposed or adopted them as being attended with almost invariable success, we are forced to the conclusion that in the ordinary run of cases constitutional treatment is of little importance. Unquestionably cases occur in which there are extreme inflammatory action and high fever, demanding the use of bleeding and antiphlogistics; and others in which the vital power is so low, and the accompanying fever assumes from the onset so marked a typhoid type, that the most powerful tonics, stimulants, and nutrients are clearly indicated. My own experience, which, however, it is right to say, has been chiefly acquired in this large and crowded city, is decidedly in favor of the tonic and stimulant plan of treatment. I ordinarily rely on the use of bark, which I give from the very commencement of the disease;—in the very old or debilitated combining it with tincture of sepentaria, as in the following form —

| | |
|---|---|
| ℞.—Tincturæ Cinchonæ, | fl ʒiv. |
| Tincturæ Serpentariæ, | fl ʒiij. |
| Tincturæ Croci, | fl ʒj. |
| Decocti Cinchonæ, | fl ℥xj. Misce. |

"An ounce to be taken every sixth hour."

At the same time giving wine and nourishing diet according to the circumstances of each case.

The preparations of iron are by many preferred to those of bark in the treatment of this disease; their mode of action would appear to be similar: the tincture of the sesquichloride, in the dose of from twenty to twenty-five drops every second or third hour, has been especially recommended by Dr. and Mr. Bell, of Edinburgh, in a paper lately published by them.*

I consider the use of purgatives in the early stages of erysipelas as decidedly objectionoble: they tend to increase the debility, usually so important a characteristic feature of danger, and, determining to the mucous membrane of the alimentary canal, and to the abdominal viscera, their action prevents the full development of the eruption on the cutaneous surface—a circumstance to be especially avoided in the treatment of all inflammatory diseases of the skin—and thus give rise to local congestions and transference of the inflammation to some internal organ.

The use of billiary evacuants has been very strongly supported in an excellent practical essay published by Dr. Albert Walsh;† the remedy he recommends being tartar emetic, in rather minute doses—one grain dissolved in a quart of some emollient drink, as whey or barley water, in the twenty-four hours; he continues its use until the eruption begins to fade, when he administers sulphate of quina and other tonics.

Mr. Lawrence is the chief advocate in the present day for bloodletting and active antiphlogistics; but in his views as regards the constitutional treatment of the disease he has very few followers.

If any internal organ be attacked during erysipelas, the most active derivatives to the surface should be employed. Thus, when the membranes of the brain are engaged, sinapisms aud blisters should be applied to the legs, and warm stupes to the head; active purgatives also are now indicated, and of these the most valuable is the turpentine enema. When the iuflammation siezes on the larynx, leeches, even although great debility be present, should be applied beneath the angles of the jaws, and hot stuping to the throat, with relays of sponges assiduously employed;

---

* Monthly Journal of Medical Science, June, 1851.

† Dublin Qnarterly Journal of Medical Science, New Series, August, 1850.

a blister to the nape of the neck is also here a valuable remedy. The operation of tracheotomy is not applicable in such cases, for the erysipelatous inflammation spreads rapidly downwards through the respirrtory tubes, causing copious effusion into their sub-mucous areolar tissue.

When erysipelas affects the scalp, the hair should be immediately cut as close as possible, with the view of keeping the surface cool, and of permitting local remedies to be more easily applied.

In the milder forms of idiopathic erysipelas, the best and only local treatment requisite consists in dusting the inflamed parts freely with wheaten flour or finely powdered starch, which may be conveniently applied from an ordinary dredging-box; the dredging should be repeated several times in the twenty-four hours. It allays the burning pain and irritation, and always proves highly grateful to the patient; therapeutically it appears to act by protecting the surface from the air, and by drying up the discharge as fast as it exudes from any vesications which may have formed. When the vesications are numerous, and the discharge excessive, I have found the addition of a drachm of oxide of zinc and twenty grains of finely powdered carbonate of lead to half a pound of starch of much advantage. In using this combination the mixed powders should be well shaken each time before the parts are dusted with them, as, in consequence of their specific gravity, the zinc and lead soon sink to the bottom of the vessel in which they are kept.

Anointing the inflamed surface with melted lard is by some preferred to the use of dusting powders. Mr. Wilson speaks highly of his experience of it, having first, he says, employed it on the recommendation of Mr. Grantham, whose method is, "to relax the skin with hot water or steam fomentations, and after each fomentation to saturate the iuflamed surface with hot lard, which is afterwards covered with wool."

When erysipelas is spreading rapidly, although superficially, over the cutaneous surface, the inflammation still persisting in the parts where it first appeared, inunction with mercurial ointment has in my experience more effect than any other local application in checking its progress. The ordinary mercurial ointment, to every once of which a drachm of glycerine has been added, should be smeared thickly over the inflamed surface, and on the sound skin for a considerable distance beyond; it need be applied only twice in the twenty-four hours, and if any symptoms of salivation be produced, its employment should be at once stopped.

Acting as an impermeable varnish, and probably producing some effect also by the compression it causes, colodion has been successfully employed by Spengler and Rapp as a local application in erysipelas; the parts are thickly coated with it by means of a camel's hair pencil, and it is renewed as often as may be required in consequence of its cracking and peeling off when dry. When the disease affects one of the extremities, bandaging the limb has been used with very favorable results: this practice originated with the Continental school: its action seems to depend chiefly on the equable compression exercised on the congested capillaries and cutaneous veins, whereby they are emptied of the excess of blood contained in them; but some of the good effect produced is also probably due to the protection from the action of the air thereby given.

Phlegmonous and traumetic erysipelas demand more active local medication than when the inflammation affects the superficial layers of the integuments merely. In these forms of the disease many rely on the topical depletion by leeches, by punctures, or by deep incisions. While leeches may produce a good effect by withdrawing blood from the inflamed superficial vessels, the determination caused to parts on which their suction power is exerted is to a certain extent productive of mischief. The samé objection does not hold with regard to punctures; their employment has been highly advocated, amongst others, by Sir Richard Dobson, by Liston, and by Wilson; they should be made with a lancet, all over the inflamed part, at distances of from a quarter of an inch to an inch, according to the extent of the surface engaged, and penetrate to the depth of a quarter of an inch. As soon as they have nearly ceased to bleed, a warm bran poultice may be applied. Mr. Copland Hutchinson strongly recommended free incisions, and his practice has been adopted by Lawrence, Guthrie, and others; they should be made down to the sub-cutaneous fascia, and be several inches in length. When there is much deep seated effusion of the liquor sanguinis, as is so frequently the case in traumatic erysipelas, they are decidedly productive of the best effect, and they should never be omitted when matter has formed.

Nitrate of silver is used in the treatment of erysipelas with two intentions,to check the spread of the inflammation superficially, and to promote resolution in the parts which have been attacked. With the former view a broad line or *cordon* is made on the sound skin, at a short distance from the margin of the inflamed surface, by the application of a solid stick of the nitrate on the part, previously wet with pure water; when the disease is situated on one of the extremities this line is made to surround the limb completely. Mr. Higginbottom, who amongst English surgeons is the chief advocate for the use of this agent, at first recommended the employment of the solid nitrate to the inflamed surface; but in his observations, recently published, and which contain the results of his accumulated experience, he states that he prefers a solution containing a scruple of the salt to a drachm of distilled water. He gives the following direction for its application —

"The affected parts should be washed well with soap and water, then with water alone, to remove any particle of soap remaining, and then wipe dry with a soft cloth: the concentrated solution of the nitrate of silver is then to be applied two or three times over the whole inflamed surface, and *beyond it* on the surrounding healthy skin, to the extent of two or three inches."

In twelve hours, should the erysipelatous inflammation be unaffected, it is to be again applied; when vesications exist, they should be opened previously to the application. I prefer to use the nitrate of silver in the form of ointment, a drachm to the ounce of lard; it thus comes more completely into contact with the inflamed surface, does not dry up so rapidly, and is more easy of application to some parts of the body, as to the scalp.

Sulphate of iron, both in solution and in ointment, has been recommended as a most valuable local application in erysipelas by Velpau, but I have not seen it prove so useful as nitrate of silver; the solution which he uses contains one part of salt dissolved in fifteen parts of water, and the ointment consists of one part of the sulphate to three or four of lard.

Blisters are sometimes employed with success to prevent the spread of erysipelatous inflammation; they are applied at the margin of the affected surface: thir effect appears to depend on a new action being excited in the parts, and their use seems to prove especially of survice in the erratic form of the disease. The only other local remedies requiring notice are, creasote, which, painted over the surface, has recently proved successful in the hands of some practitioners; and congelation of the surface by means of pounded ice and salt mixed in a bladder, which has been proposed by Dr. Arnott: from the use of the latter it may be apprehended that some internal organ might be attacked.

Should erysipelas assume a gangrenous tendency, in addition to the internal administration of the most powerful tonics, as wine, bark, and quina, the parts ought to be enveloped with a charcoal poultice, and afterwards dressed with lint soaked in a lotion, containing from one to two ounces of the solution of chlorinated soda or chlorinated lime to the pint of distilled water.

The Vesiculæ form an interesting class of cutaneous disorders. Since the time of Willan, they have been restricted to those forms in which "the effusion is a transparent fluid, contained in minute, orbicular, epidermic elevations." The fluid, in the progress of the disease, becomes opaque, and dries into thin scales or crusts. Of the first variety included under this head our author remarks—

Eczema (Scall or Humid Tetter) is a most important and interesting disease of the skin, being of extremely frequent occurrence, at times very difficult of diagnosis—particularly in its advanced stages, and usually most rebellious to treatment. It is characterized by the eruption of numerous minute transparent vescicles, closely set and irregularly aggregated on an uncircumscribed inflamed surface, and attended generally with burning pain and intense itching. It is highly inflammatory, but not contagious. The vesicles, which are at first perfectly transparent become opaque on the second or third day after their appearance—the contained fluid assuming a semi-purulent character—and either dry up with a fine furfuracious desquamation, or bursting, become covered with thin, yellow crusts, from beneath which an acrid, watery exudation takes place.

When it assumes a chronic form, it is a most intractable eruption. Dr. Neligan mentions a case in which it persisted for twenty-five years; and according to his experience it is rarely cured under several months' treatment. The following is what he says on the subject of treatment—

Eczema is essentially an inflammatory eruption, even in its most chronic stages, and this fact should always influence our choice of remedies, whether topical or constitutional, for its treatment. In eczema simplex, occurring in adults, mild saline antiphlogistics with alkalies, as in the following form, are prescribed with advantage at its commencement—

℞.—Sodæ et Potassæ Tartratis, ℥j.

Solutionis Alkalinæ (Brandish), fl ʒiv.

Aquæ destillatæ, fl ℥xixss. Misce.

"A wine glass full to be taken three times a day."

For children, gentle mercurial purgatives are better adapted, combined with antimonials, such as James' powder, if the febrile symptoms are well marked. In the mild forms of the disease the best local treatment in the acute stage is the use of the general tepid bath, or of warm water sponging, the parts being thoroughly but gently dried afterwords. When the local affection is more severe, the weak lead-wash—a drachm of the solution of subacetate of lead to twelve fluid ounces of rose or elder-flower water—applied on old linen wet well with it, is an excellent application: if the eruption is seated on any part of the extremities, it is best and most efficiently applied by means of bandages evenly put on, and kept constantly moist with the wash. In some cases of eczema, moisture appears to disagree singularly, always aggravating the local symptoms; under such circumstances an ointment containing four grains of the carbonate of lead, or of the acetate of zinc, to an ounce of cold cream, may be used, and if there is much tingling or itching in the part, two minims of prussic acid should be added to the latter, or six minims of chloroform to the former ointment. If simple exzema occurs, as is not unfrequently the case, in children of a scrofulous diathesis, it is very apt to become chronic; when it does so, local remedies seem to have little effect on the eruption, but it yields rapidly to the internal administration of cod liver oil, and the daily use of the tepid fresh-water bath.

In the early stages of eczema rubrum general antiphlogistic treatment, proportionably active according to the inflammatory character of the constitutional and local symptoms is requisite; in persons of full habit of body bleeding from the arm even may be indicated, and in most cases topical bleeding by leeches from the neighborhood of the affected parts is attended with much benefit; active saline cathartics should be administered and repeated at short intervals, until the febrile symptoms are subdued. The local heat, swelling and tingling are best alleviated by gelatine baths, and at night the application of poultices, prepared by first steeping the best white bread in boiling water, squeezing it out as dry as possible, and then moistening the pulp with the weak lead-wash above mentioned.

When this form of disease becomes chronic, it is usually most rebellious to treatment, and requires the employment of internal specific or alterative medicines to produce a change in the state of the constitution with which it is combined, or on which it may depend, and this is requisite even though the eruption is of small extent and local. In many cases the preparations of iodine prove most efficacious, but sometimes it is requisite to combine them with arsenic, or to give that medicine alone; in delicate constitutions, or if debility be present, the iodide of potassium is the best form; it may be given in some tonic decoction, as follows —

℞.—Iodidi Potassii, gr. viij.

Decocti Ulmi (corticis recentis, fl ℥xij.

Decocti Dulcamaræ, fl ℥iv. Misce.

"A wine-glass full to be taken every night at bed time."

When thus given it is not liable to sicken the stomach, and in my experience small doses of iodine or its preparations act most efficaciously in the treatment of diseases of the skin. If the use of arsenic be indicated, either from the obstinacy of the affection or the failure of other remedies, five minims, very gradually increased to eight, of the liquor potassæ arsenitis, or of the liquor arsenici chloridi of the last London Pharmacopœia, may be added to each dose of the above mixture; but whether iodine or arsenic be administered alone or in combination, their use must be continued for a long time, at least for two or three months, and beneficial results are always most effectually derived from the system being brought very gradually under their influence. Numerous internal medicines, as well as local applications, have been recommended by different writers for the treatment of chronic eczema; of the former, the most generally employed are the tincture of cantharides, antimonials, sulphur especially in the form of the sulphurous mineral waters, mercurials, and various vegetable tonics; but my own experience leads me to rely on either or both of the powerful alteratives above recommended; even in the most chronic cases I have seen the sulphurous waters—so valuable in other diseases of the skin—prove injurious, and mercurials generally disagree except with children, in whom the green iodide of mercury, combined with the hydrargyrum cum creta, often acts as a most valuable alterative.

The itching and copious secretion attendant on chronic eczema demand the employment of local sedative and astringents. The compound lead cerate of the London Pharmacopœia, to every ounce of which two drachms of glycerine and eight minims of chloroform have been added, constitutes a most useful ointment, no matter on what part of the surface the eruption may be seated; or the carbonate of lead or acetate of zinc ointment already described may be substituted for it should there be much tendency to local inflammatory action. Tannic acid, in the proportion of from four to twelve grains to the ounce of cold cream, with or without the addition of chloroform, is also an excellent application. Some recommend highly stimulating compounds for the local treatment of chronic eczema, such as anthrakokali or fuligokali (forms of carburet of potassium, which, when introduced into the practice of medicine a few years since, were highly vaunted for their remedial powers, but have now fallen into disuse) tar, pitch, sulphurous preparations, etc. By distilling tar with water, a mixture of impure oil of turpentine, a pyrogenous oil, and some pyretine is procured ; this liquid has been recently used and highly praised by some French dermatologists, under the name of *huile de cade*, as a local application, inunctions being made with it twice daily; by the term *huile de cade*, however, most of the French pharmacologists understand a tarry oil obtained by the dry distillation of the wood of the juniperus oxycedrus. No matter what local remedy is employed, it will be found of advantage to sponge the affected parts carefully with a *weak* alkaline wash—ten grains of the carbonate of soda to a pint of distilled water, each time previously to its fresh application.

When eczema occurs on the face or hands, the treatment as above described for its different forms is equally applicable, but in either situation, in consequence of the exposure to atmospheric vicissitudes and to various local irritants, it is usually more obstinate, and when the disease is general over the body the face is for the same reasons the last part to get well. The use of soap in washing should be interdicted, the weak carbonate of soda lotion warmed to blood heat being substituted for it, and especial care should be taken not to expose the surface to the action of harsh winds, the sun, the heat of the fire, or any cause which might produce determination of blood to the affected parts.

In eczema capitis the hair should be cut close to the scalp with a sharp pair of scissors—not *shaved* off—and kept as short as possible while the disease lasts, and for a short time after it is apparently cured. The crusts and scabs should be removed by poulticing with linseed meal, and sponging with the weak carbonate of soda solution mixed with an equal quantity of new milk, the surface being carefully dried afterwards. When the scalp is thus cleaned, in the milder and less inflammatory forms of the eruption an alkaline ointment, containing twelve grains of the bicarbonate of soda to the ounce of cold cream, may be applied morning and evening, the surface having been previously sponged as above directed; but if there be any tendency to inflammatory action, the carbonate of lead or the tannic acid ointment, and the subacetate of lead-wash, prove more beneficial. The scalp should be kept cool, very lightly covered or exposed to the air when in the house. As regards constitutional treatment, in scrofulous habits of body, either cod liver oil or iodide of potassium, with tonics, should be administered; but the best alterative for children who are not scrofulous is the green iodide of mercury, as before mentioned.

In all forms of eczema, when the origin of the disease can be traced to any local irritant, this, of course, should be carefully guarded against, both during the progress of treatment and when a cure is affected; in the case of persons engaged in trades, and others who cannot abandon the occupation by which the eruption was caused, the cutaneous surface should be protected as much as possible by the use of wash leather gloves. The diet of persons affected with eczema should be regulated according to the constitutional circumstances, but in children much benefit will be derived from placing them on a strictly milk and farinaceous diet. In both adults and children the bowels require to be carefully attended to, the use of mild saline purgatives, if possible the natural mineral waters,of which probably the Pullna is the best, being employed with excellent effects. In children the process of teething must be watched, and the gums lanced when necessary; and the eruption on the scalp should not be dried up too suddenly if there exists a tendency to disease of the brain, or any other internal organ.

We have preferred to give a few long extracts from this valuable little treatise, rather than to attempt an analysis of its contents, as affording the reader a better opportunity of forming a correct estimate of its merits. It strikes us as being just such a manual as the practitioner would like to possess on diseases of the skin. As a summary of professional experience on this interesting class of diseases it is judicious and eminently satisfactory. Among the elaborate and costly works issued on the subject, the publishers have shown a correct appreciation of the wants of the profession in bringing out this cheaper publication. We are confident it will find many readers.

